# All-Atom Molecular Dynamics Simulations of Grafted Poly(*N*,*N*-dimethylaminoethyl methacrylate) Brushes

**DOI:** 10.1021/acs.jpcb.4c07928

**Published:** 2025-02-10

**Authors:** Simon Tippner, David Hernández-Castillo, Felix H. Schacher, Leticia González

**Affiliations:** † Institute of Theoretical Chemistry, Faculty of Chemistry, University of Vienna, Währinger Straße 17, 1090 Vienna, Austria; ‡ Doctoral School in Chemistry (DoSChem), University of Vienna, Währinger Straße 42, 1090 Vienna, Austria; § Laboratory of Organic and Macromolecular Chemistry (IOMC), Friedrich Schiller University Jena, Humboldtstraße 10, 07743 Jena, Germany; ∥ Vienna Research Platform on Accelerating Photoreaction Discovery, University of Vienna, Währinger Straße 17, 1090 Vienna, Austria

## Abstract

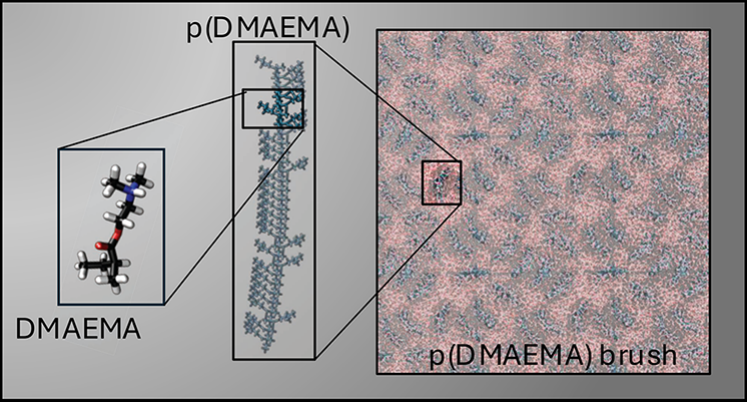

Modeling polymer brushes is essential for understanding their complex behavior at surfaces and interfaces, enabling the design of materials with tunable properties. We present a computational protocol to model polymer brushes composed of grafted, brush-like chains of the charged polymer poly(*N*,*N*-dimethylaminoethyl methacrylate) (p(DMAEMA)) using an all-atom representation that captures detailed molecular interactions and structural properties. The approach is flexible and non-grid-based and allows for randomized strand configurations and the incorporation of periodic boundary conditions, enabling the construction of asymmetric polymer brush setups. An atactic p(DMAEMA) configuration is demonstrated as an example, though the protocol can be readily adapted to construct other brush-like polymer systems with varying tacticities or compositions, depending on the pH environment. Furthermore, this can be extended to stimuli-responsive materials, which generate conformation or charge upon changes in pH value or other external triggers. Molecular dynamics simulations are then employed to gain insights into the conformational behavior of the grafted p(DMAEMA) brushes and their surrounding aqueous environment, as well as their response to temperature, protonation, and variations in grafting densities, in terms of the solvent-accessible surface area, radius of gyration, and radial distribution functions. This versatile protocol provides a robust tool for simulating and analyzing the properties of diverse polyelectrolyte polymer brush systems and also as composite materials.

## Introduction

Polymer brushes are macromolecules where polymer chains are grafted to an interface or to the backbone of another polymer.^1^ By tailoring polymer characteristics, such as grafting density, chemical composition, charge and electrolyte composition, along with the surfaces or substrates where the polymers are tethered,^2,3^ various interesting properties and functionalities can be engineered. As a consequence, grafted polymers are used in a wide range of applications; these include serving as building blocks for nanostructures such as polymer-grafted nanoparticles,^4,5^ enhancing the biocompatibility of nanomaterials,^6^ or improving the performance of energy devices when integrated into nanoarchitectures.^7^ A widely studied subclass of polymer brushes is polyelectrolyte polymer brushes,^8−10^ which are composed of charged polymer chains. These materials allow to introduce responsive materials to various environments and control over conformation, net charge, and charge density by external parameters.

The combination of soft and hard materials, commonly categorized as organic and inorganic components, respectively, opens up possibilities for creating novel composites with unique properties that would otherwise be unattainable.^11,12^ The key advantage of such combinations lies in the synergy between the mechanical strength provided by the hard component and the flexibility inherent in soft-matter structures. This merging offers additional beneficial features such as structural stabilization and protection.^13^ Polymers, as a promising class of soft matter, have shown great potential in the area of composites, also with regard to block copolymers,^14^ which allow chemical linkage of different polymers to form innovative structures with complementary properties of organic (soft) and inorganic (hard) matter.

**Figure 1:**
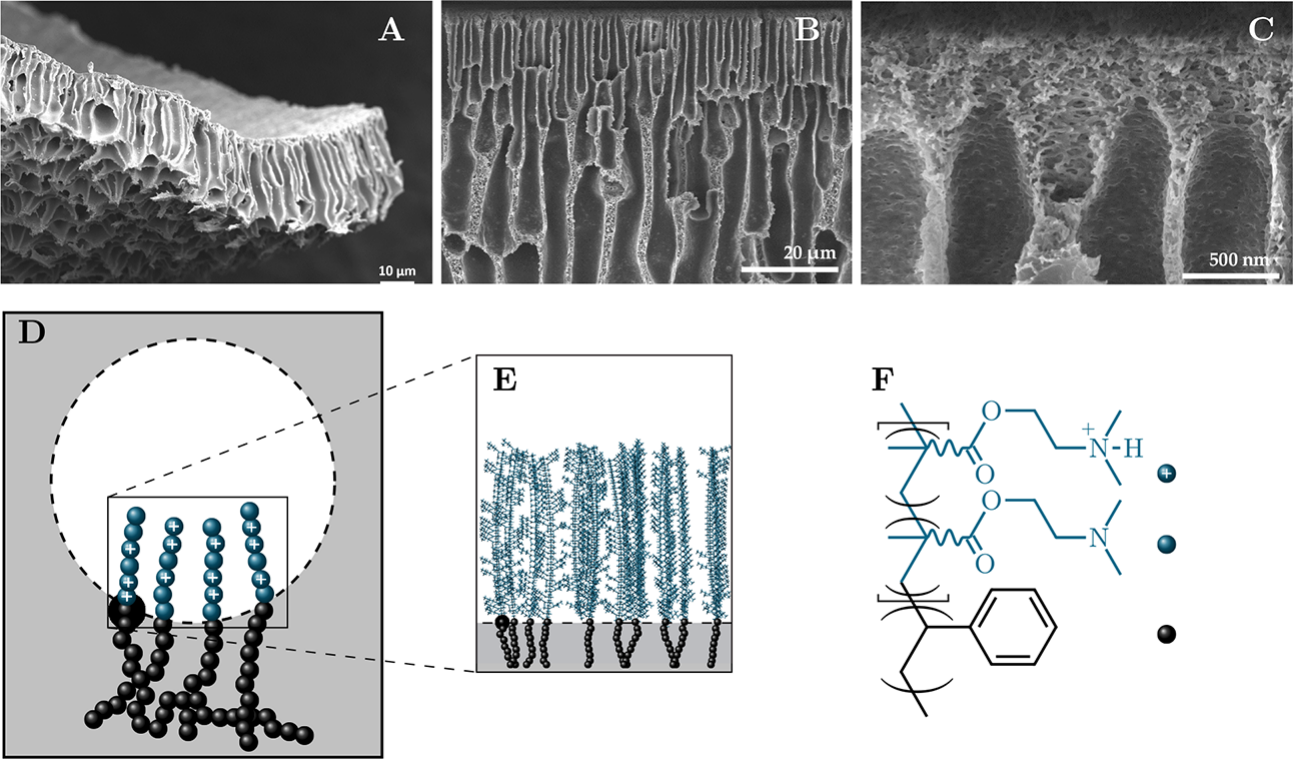
(A–C) Scanning electron microscope micrographs of a PS-*b*-p(DMAEMA) block copolymer membrane prepared via nonsolvent induced phase separation: (A) cross sectional overview; (B, C) higher magnifications of the cross section (details regarding membrane preparation are published elsewhere,^17^ details regarding the scanning electron microscope measurements are provided in Section S1.); (D) schematic representation of the block copolymer chain within the pore of such a membrane, with the hydrophobic polystyrene units (black beads) that form the pore wall and the DMAEMA units (blue beads) tethered to it; (E) inset with the pore wall simplified as flat and p(DMAEMA) brushes (in blue); (F) chemical structure of PS-*b*-p(DMAEMA).

Organic–inorganic composites are valuable in catalysis, as soft-matter matrices can provide superior environments for different catalysts and prolong the catalyst's lifetime or control substrate diffusion. Thus, by integrating catalysts into a polymeric surface, it becomes possible to actively control the chemical environment, optimizing both catalyst activity and stability.^13^ Additionally, this approach can enable high catalytic performance while allowing for rapid and efficient catalyst recovery.^15^ In this context, polymer brushes and their hybrid combinations with inorganic materials offer versatile platforms for engineering advanced materials with tailored functionalities for a variety of applications.^16^ One recent example is the sponge-like nanoporous polystyrene-*block*-poly(*N*,*N*-dimethylaminoethyl methacrylate) (PS-*b*-p(DMAEMA)) block copolymer membranes, where the hydrophobic PS forms the membrane matrix and the hydrophilic p(DMAEMA) covers the membrane surface (see Figure 1).^17^ Such charged p(DMAEMA) chains can be used for the immobilization of polyoxometalate catalysts and/or photosensitizers by electrostatic interactions, resulting in hybrid materials that are both capable of light-driven proton reduction and water oxidation.^17,18^

The polymer chain p(DMAEMA) contains one amino group per DMAEMA unit that can be either protonated or unprotonated, depending on the system’s pH.^19^ Additionally, the polymer chain p(DMAEMA) exhibits stereochemistry due to its chiral methacrylate units. Due to that chirality, p(DMAEMA) shows tacticity. The term tacticity was coined by the Italian scientist Giulio Natta and characterizes the arrangement in which monomeric units follow one another along the chain with steric configurations.^20^ Three types of tacticity are commonly distinguished: (i) Isotactic polymers are those where the repeating unit configuration is consistent along the chain, meaning that the substituent groups or side chains are aligned on the same side of the polymer backbone. This results in a highly ordered and stereoregular structure. (ii) Syndiotactic polymers display units so organized that, moving from one unit to the next along the chain, there is an inversion in the steric configuration. This results in alternating stereoconfiguration throughout the polymer chain. (iii) Finally, atactic polymers lack any steric order or tacticity, featuring randomly distributed substituent groups or side chains along the polymer backbone, which results in an irregular structure and a generally amorphous nature.^20,21^ The tacticity of polymers can be influenced experimentally using catalysts, temperatures, or polymerization techniques.^22^

Rationalizing the behavior of highly versatile materials such as the PS-*b*-p(DMAEMA) block copolymer requires a precise atomistic understanding of their response to stimuli. In this regard, computational modeling at the atomistic level has become an indispensable tool to understand macromolecular behaviors,^23,24^ such as swelling or collapse of polymer brushes observed in experiments. All-atom models, despite being more expensive, are better than coarse-graining representations^25^ to capture mechanistic details of the structural and thermodynamic properties of polymer brushes.^26,27^ Previous research on p(DMAEMA) has explored the coil-to-globule transition of single polymer threads using all-atom molecular dynamics (MD) simulations, offering insights into the temperature-dependent conformational behavior of the polymer.^28^ Mintis et al.^29^ have investigated the impact of applying various force fields to accurately describe the conformational and dynamic properties of p(DMAEMA) in solution, comparing these results with experimental data across different polymer concentration regimes. Both studies share the use of alternately protonated atactic p(DMAEMA) chains. Fully atomistic simulations of p(DMAEMA) polyelectrolyte brushes were performed by Santos et al.,^30^ describing the swelling behavior of the brushes in response to changes in solvent, pH, and salt types. In their model, they applied a randomly chosen protonation/deprotonation set. They achieved brush confinement by covalently attaching each polymer chain to a thiol initiator and positionally restraining the carbon atoms during the simulation to represent the substrates’ surface. However, information about the tacticity of the polymer as well as the position of the grafted polymer threads was missing. Extensive research has been conducted on various types of MD simulations of polymer brushes. The majority of these studies utilized a fixed grid where polymer strands were grafted onto a plane,^31−34^ even if this method does not capture the natural irregularity of the brush surface.

Given the diversity of approaches and the absence of a detailed, user-friendly roadmap about how to construct polyelectrolyte brushes for fully atomistic MD simulations, in this paper, we report a computational protocol to generate atactic p(DMAEMA) polyelectrolyte brushes using an all-atom representation that allows capturing every molecular interaction and structural property. p(DMAEMA) is used as a model system to enable a better understanding of the behavior of such hybrid materials and how varying external parameters might affect the stability of any immobilized catalyst, the accessibility of active sites, or eventual leaching if such membranes are used under flow-through conditions. Our protocol can nevertheless be easily adapted to construct other brush-like polymeric systems with different tacticities in different pH environments. Here, all-atom MD simulations are used to gain insights into the conformational behavior of the p(DMAEMA) brushes and their surrounding solvent environment and how they are influenced by variations in grafting densities, temperature, and pH.

## Computational Setup

**Figure 2:**
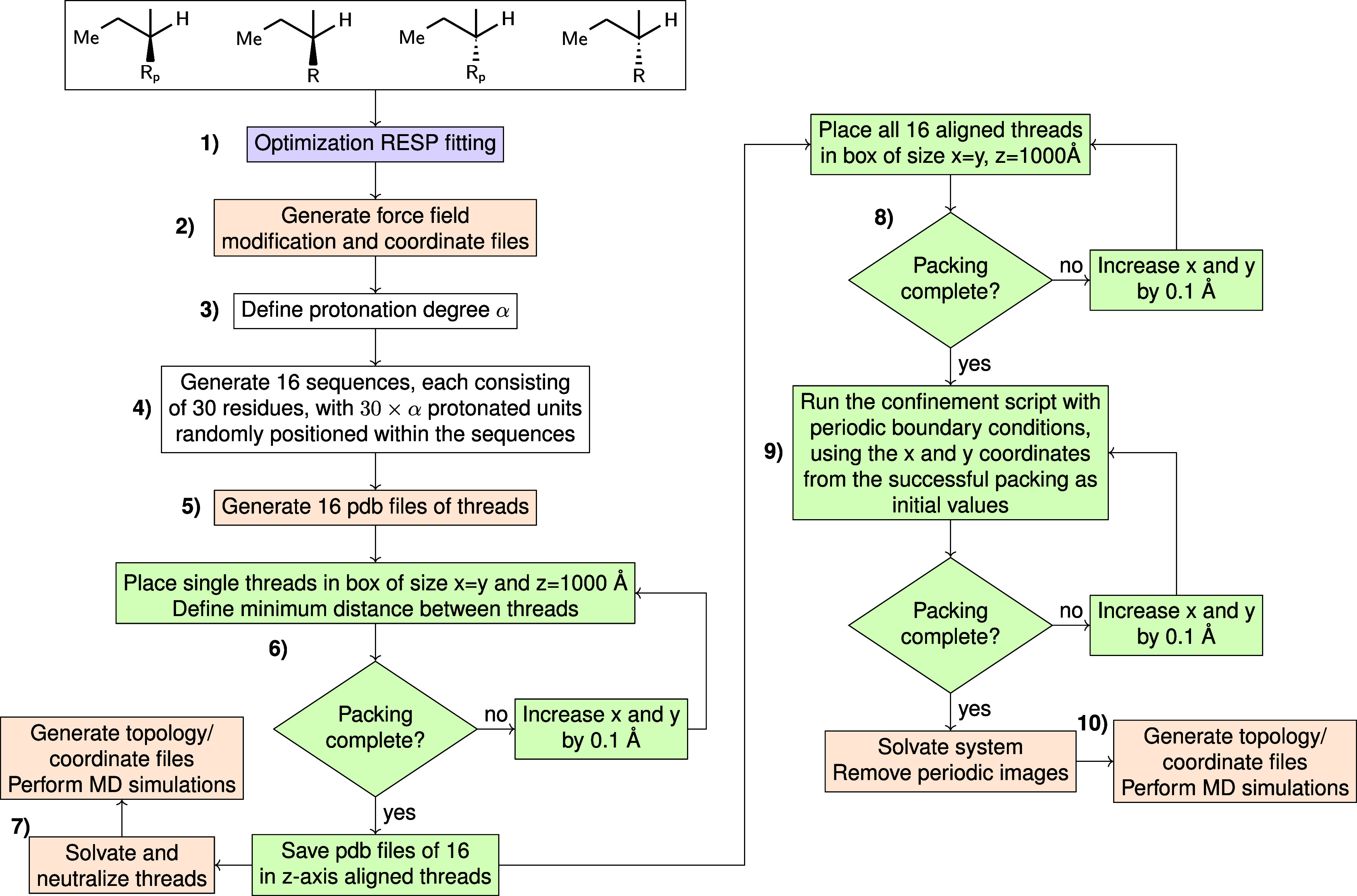
Schematic flowchart illustrating the workflow of the grafting process for individual p(DMAEMA) threads, beginning with the DMAEMA monomers. The various software packages utilized are indicated by colors: Gaussian (violet), Amber (orange), and PACKMOL (green).

This section describes the construction of polymer brushes. The entire procedure is illustrated in the flowchart depicted in Figure 2, where key steps are numbered to guide the reader through the process. We begin by building a single DMAEMA unit from which multiple atactic 30-mer threads (p(DMAEMA)) are stochastically generated. A chain length of 30 monomers is selected because it provides sufficient length to effectively demonstrate polymer’s phase transition behaviors.^29^ Subsequently, 16 of these chains are then confined to create a polymer brush.

### Generation of p(DMAEMA) Threads

**Figure 3:**
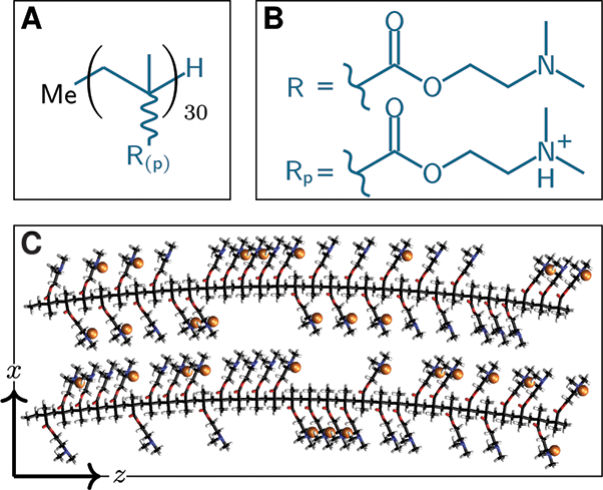
(A) Schematic chemical structure of 30-mer p(DMAEMA). R_(p)_ stands for the 2-(dimethylamino)ethyl side units R, which can be protonated (p) or not. (B) Chemical structures of the 2-(dimethylamino)ethyl side chain, one protonated (R_p_) and one unprotonated (R). (C) Two representative structures of atactic p(DMAEMA) 30-mer strands with a protonation degree α of 0.5. The orange beads indicate that the proton is attached to the nitrogen atoms of the protonated units.

Each p(DMAEMA) is constructed from 30 DMAEMA units that can adopt any of the four configurations depicted in Figure 2. The polymer features a fully extended carbon backbone that, for the sake of computational efficiency, is built with a methyl group terminating at one end (instead of the PS block of Figure 1) and a hydrogen atom at the other; see Figure 3A. The stereoconfiguration of the side chain R_(p)_ of the DMAEMA monomer is indicated by the wavy bond in Figure 3A. Figure 3B presents the structure of the 2-(dimethylamino)ethyl side chain in both protonated and unprotonated forms. To quantify the number of protonated DMAEMA units, we define the protonation degree α as the ratio of R_p_ to R. By changing the protonation degree, we are able to mimic different pH conditions of the system. Through potentiometric titration measurements, Lee et al.^19^ investigated the pH-dependent protonation behavior of p(DMAEMA). They determined the p*K*_a_ of p(DMAEMA) to be approximately 7; however, this value is dependent on experimental parameters such as the polymer molecular weight and concentration. Following this, at a protonation degree of α = 0.5, we assume pH = p*K*_a_. In our study, we distinguish three scenarios: When α = 1.0, all 30 units are fully protonated, which we associate with an acidic pH range; when α = 0.5, 15 units are protonated, and we assume a neutral pH range. Finally, when α = 0.0, no units are protonated, corresponding to a basic pH range. For α = 0.5, the positions of the protonated units were randomly distributed across all structures.

To enable the generation of the 30-mer p(DMAEMA) threads, we start with the two stereoisomers of the DMAEMA monomer in their protonated MeCH_2_CHR_p_CH_3_ and unprotonated MeCH_2_CHRCH_3_ form, respectively. All four structures were optimized using density functional theory, as described in the Computational Details. The chemical structures are shown in the flowchart in Figure 2. The electronic densities obtained from these calculations were then used to generate the partial atomic charges for all four residues based on the restrained electrostatic potential method (Figure 2, Step 1).^35^Table S1 displays the Coulomb partial charges on all atoms of the p(DMAEMA) chain. To establish connectivity between the residues, we defined main chain topology files. These files include information about how adjacent residues are linked. From those four structures, we defined three residues, respectively: the initial (MeCH_2_CR_(p)_CH_3_−), middle (−CH_2_CR_(p)_CH_3_-), and terminal (−CH_2_CHR_(p)_CH_3_) groups, resulting in a total of 12 different residues. Using the antechamber tool within the AMBER software suite,^36^ the force field modification files and coordinate files —which include the restrained electrostatic potential charges— were created for all residues (Figure 2, Step 2). A script was developed to produce a random and atactic string composed of 30 residues. It allows the user to freely choose the protonation degree (Figure 2, Step 3). In our case, we considered a protonation degree of α = 0.5, resulting in 15 protonated and 15 unprotonated DMAEMA residues. A total of 16 random strings were generated (Step 4). Subsequently, these randomly generated strings, along with the modification and coordinate files, were utilized to create the PDB files through tleap, a tool implemented into the AMBER software suite (Step 5). Figure 3C displays two representative structures of the atactic p(DMAEMA) strands. While the number of protonated units is consistent between them, their positions differ. These geometries, generated using tleap, were initially unoriented in space. To enable subsequent confinement into a polymer brush, we aligned each strand along the *z*-axis using the PACKMOL software,^37^ which facilitates molecular grafting within a confined space. Each polymer strand was then placed in a box with dimensions *x* = *y*, chosen such that PACKMOL is unable to enclose the single thread within the box. This procedure “forces” the polymer to align in the *z*-direction, which is arbitrarily set to a high value of 1000 Å. If PACKMOL was unable to pack the strand inside the box, then both the *x* and *y* values were incremented by 0.1 Å, and the grafting process was reiterated. This procedure continued until each polymer strand fits within its respective box. Step 6 of the flowchart in Figure 2 provides a schematic explanation of the loop. Using this approach, we guarantee the alignment of each polymer strand in the *z*-direction. This step is advantageous for the subsequent construction of the polymer brush, with every thread aligned along the *z-*axis. The procedure was performed for all 16 atactic 30-mer p(DMAEMA) strands, each with a protonation degree of α = 0.5 and different positions of the protonated units. Fully protonated (α = 1.0) or unprotonated (α = 0.0) strands were produced by adding or removing hydrogen atoms from the nitrogen atoms within the set of 16 semiprotonated p(DMAEMA) threads. These generated polymer strands were subsequently used to construct the brush-like p(DMAEMA) polymer brush. To prepare the individual polymer threads for performing the MD simulations, we placed each of them in a 73 × 73 × 142 Å^3^ simulation box filled with water, ensuring a water density of 1 g/cm^3^. This box size proved to be adequate to prevent any interaction with its periodic images, as the goal was to investigate the polymer threads individually. The system was neutralized by adding the appropriate number of chloride ions: 0 for α = 0.0, 15 for α = 0.5, and 30 for α = 1.0. After the grafting and solvation steps were finished, each of the 16 threads was subjected to MD simulations (Figure 2, Step 7), performed as described in Computational Details.

### Formation of a p(DMAEMA) Polymer Brush

**Figure 4:**
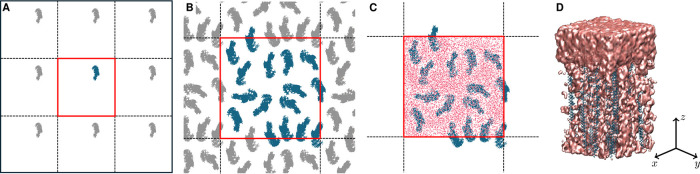
Confinement process for 16 30-mer p(DMAEMA) strands utilizing periodic boundary conditions. (A) Placement of a single polymer strand (blue) in a central box (red) with its 8 periodic images (gray) around. (B) Top view showing all 16 strands, including periodic copies. (C) Solvated box containing all 16 p(DMAEMA) threads, excluding the periodic copies, prepared for the subsequent MD simulation. (D) Side view of the full-atom p(DMAEMA) polymer brush.

After all 16 atactic 30-mer p(DMAEMA) threads (α = 0, 0.5, and 1.0) were generated and aligned in the *z*-direction, they were confined to form a polymer brush. First, similar to the alignment of the single threads, all 16 polymer strands were enclosed within a square box with dimensions (*x*, *y*, *z*), where *x* = *y* and *z* was again set to an arbitrarily large value of 1000 Å, using PACKMOL. Rotations were allowed around the *z-*axis. The lowest carbon atom of each thread was anchored onto the *xy* plane to ensure a consistent height profile throughout the polymer brush. The *x* and *y* dimensions were selected to result in an insufficient box size such that PACKMOL was not able to successfully pack the 16 threads in the box. Iteratively, these dimensions were incremented by 0.1 Å until a sufficient box size (with *x*_init_ and *y*_init_) was achieved to confine all 16 threads within the given box. Step 8 of the flowchart in Figure 2 provides a schematic explanation of the loop. Because at the time of working, PACKMOL did not have periodic boundary conditions implemented, we simulated them manually as follows (Step 9). Two boxes were defined: one with dimensions *x*_init_ and *y*_init_ obtained from the previous confinement procedure of the 16 threads, and a second, larger box with dimensions three times greater, enclosing the smaller box and centered around it, as shown in Figure 4A. The smaller box is depicted in red, while the larger box is shown with solid black lines.

**Figure 5:**
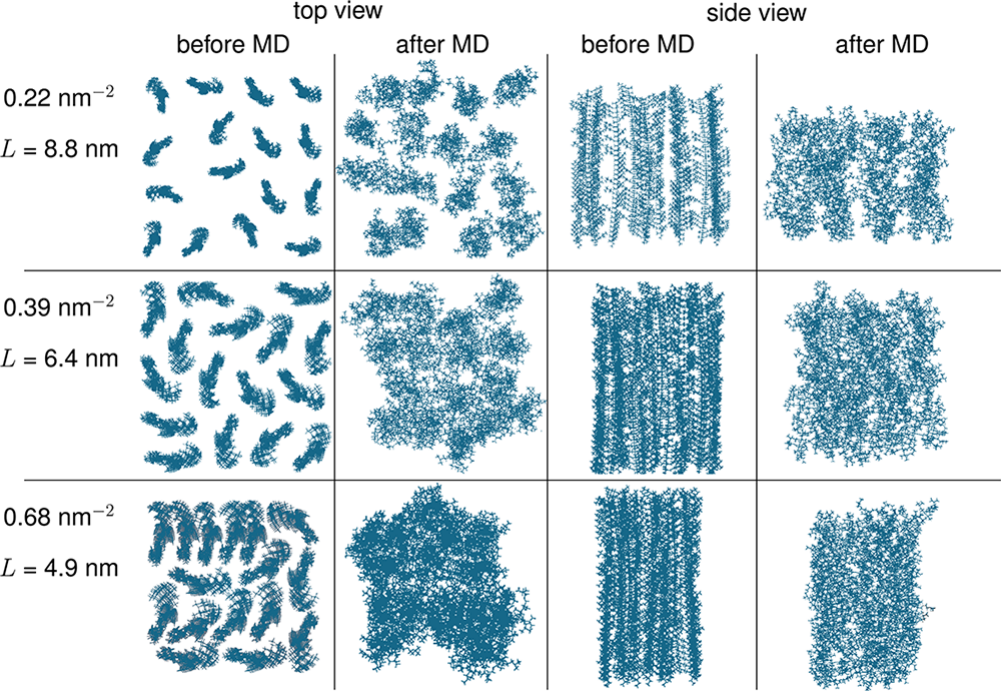
Top and side views of three p(DMAEMA) polymer brushes with varying grafting densities, before and after the MD simulation. Water molecules are removed for clarity.

In black dashed lines, we illustrate eight boxes surrounding the central red box. These boxes represent the periodic images. In the first step, utilizing PACKMOL, a p(DMAEMA) strand was randomly positioned within the red center box, fixing its lowest carbon atom on the *xy* plane. Subsequently, this thread was duplicated into the periodic boxes by adding or subtracting *x*_init_ and *y*_init_ to the coordinates of the previously positioned thread. The periodic p(DMAEMA) threads are colored in gray. Following that, the second thread was positioned within the central box, enabling it to experience the influence of the previously confined thread plus its periodic copies. Once more, the newly placed polymer strand was replicated across its periodic images. This process was iteratively repeated. At each step, if a thread confinement attempt failed three times, the process was restarted from the grafting of the first thread. If the entire process failed 300 times, the box dimensions were considered too small and were increased by 1 Å. The procedure was repeated until all 16 threads fit within the box (Figure 4B). This method ensures dense grafting while accounting for the periodic boundary conditions. Utilizing this process, we generated three p(DMAEMA) brushes with various densities of 0.68, 0.39, and 0.21 p(DMAEMA) threads per nm^2^. Finally, the entire system, including the periodic images, was solvated with water, ensuring a water density of 1 g/cm^3^. Above each polymer brush, a water layer of 30 Å was added in order to prevent interactions between periodic images of the brushes along the *z* direction. Water molecules residing outside the central box were removed, along with all periodic images of the threads (Figure 4C). As an example, Figure 4D depicts a p(DMAEMA) polymer brush from the side. Subsequently, the system was neutralized by adding chloride ions to the whole water box and subjected to MD simulations (Figure 2, Step 10), performed as described in Computational Details. This procedure provides a method for grafting the polymer threads onto a plane using a non-grid approach, which randomizes the positions of individual polymer threads. Figure 5 shows the initial structures of the constructed p(DMAEMA) polymer brushes with varying grafting densities and the specific box lengths *L* (= *x* = *y*).

## Computational Details

### Quantum Chemical Calculations

The electronic densities for fitting of the restrained electrostatic potential charges were obtained from geometry optimizations of the DMAEMA units (protonated/unprotonated) with density functional theory, performed at the B3LYP/def2-SVP level of theory,.^38−41^ Dispersion interaction effects were included using Grimme’s D3 model with Becke-Johnson damping.^42,43^ These quantum chemical calculations were carried out with the Gaussian 16 software.^44^ The specific values of the partial atomic charges can be found in Table S1.

### MD Simulations

All the MD simulations were performed using the Amber22 Molecular Dynamics Package.^45^ To describe p(DMAEMA), the Generalized Amber Force Field (GAFF2) was used.^36^ A total of two sets of 16 p(DMAEMA) threads with a protonation degree of 0.5 were constructed, differing in their stereochemistry as well as the position of protonated units. Fully protonated and deprotonated strands were constructed by adding or removing hydrogen atoms from the respective units. Each p(DMAEMA) polymer brush with the same grafting density was constructed with each set of 16 p(DMAEMA) threads (α = 0.5) according to the procedure described in Figures 2 and 4, accounting for different grafting geometries. The single p(DMAEMA) threads were solvated with a total of 25,133 water molecules. The three different polymer brushes, with grafting densities of 0.68, 0.39, and 0.21 nm^–2^ were solvated with 2740, 8526, and 21,253 water molecules, respectively. Initially, the solvent was minimized within 10,000 steps by applying a positional restraint of 500 kcal/(mol Å^2^) to the solute. Afterward, all restraints were removed, and the entire system was minimized for an additional 10,000 steps. In both minimizations, we employed the steepest gradient algorithm for the first 5000 steps, followed by the conjugate gradient algorithm for the last 5000 steps. Once energy minimization was completed, the system was rapidly heated to 100 K within 5 ps (2500 steps) and then to 300 K over 100 ps (50000 steps). The Langevin thermostat and periodic boundaries for a constant volume were enabled. During the heating step, a restraint of 10 kcal/(mol Å^2^) was applied to the solute. Subsequently, constant pressure conditions were used to allow the density to relax. Two short NPT simulations of 20 ps each were performed, followed by a longer simulation of 2 ns. Finally, a long NPT simulation of 200 ns was carried out. In all NPT simulations, a restraint of 10 kcal/(mol Å^2^) was applied to the lowest residues of the p(DMAEMA) strands, fixing them on the same plane, and isotropic pressure was utilized. All MD simulations of every system were performed four times with random starting velocities and periodic boundary conditions enabled in all directions. A total of 128 MD simulations were conducted for the single p(DMAEMA) threads. This number comes from a combination of factors: 2 different sets of 16 unique p(DMAEMA) threads and 4 individual simulation runs per thread (2 × 16 × 4). A total of 24 MD simulations were conducted for the polymer brushes, with four simulations performed for each polymer brush at a distinct grafting density, constructed from the two sets of individual p(DMAEMA) threads. Time steps of 2 fs were applied with the SHAKE algorithm to preserve the bonds and angles of water molecules.^46^ A cutoff for nonbonded interaction terms was set to 10 Å. The MD simulations were accelerated by using the GPU (CUDA) version of pmemd.^47−49^

## Results and Discussion

In the following, the conformational behavior of the p(DMAEMA) brushes and its response to the water environment are analyzed with MD simulations.

### Radius of Gyration (ROG) and Solvent-Accessible Surface Area (SASA)

The ROG is defined as the average distance between the monomers of the polymer and the center of mass of the polymer, effectively characterizing the compactness of the molecule in space.^50^ A larger value indicates a more extended chain, while a smaller ROG represents a more coiled configuration. The SASA provides information on the total surface area of a molecule that is accessible to solvent molecules.^51^ It indicates how much of the molecule’s surface is exposed to the surrounding environment, which is essential for understanding chemical interactions. A probe of van der Waals radius of 1.4 Å was used to determine the surface area. Both ROG and SASA values for the single 30-mer p(DMAEMA) strands were calculated using the AMBER built-in tool CPPTRAJ.^52^

**Figure 6:**
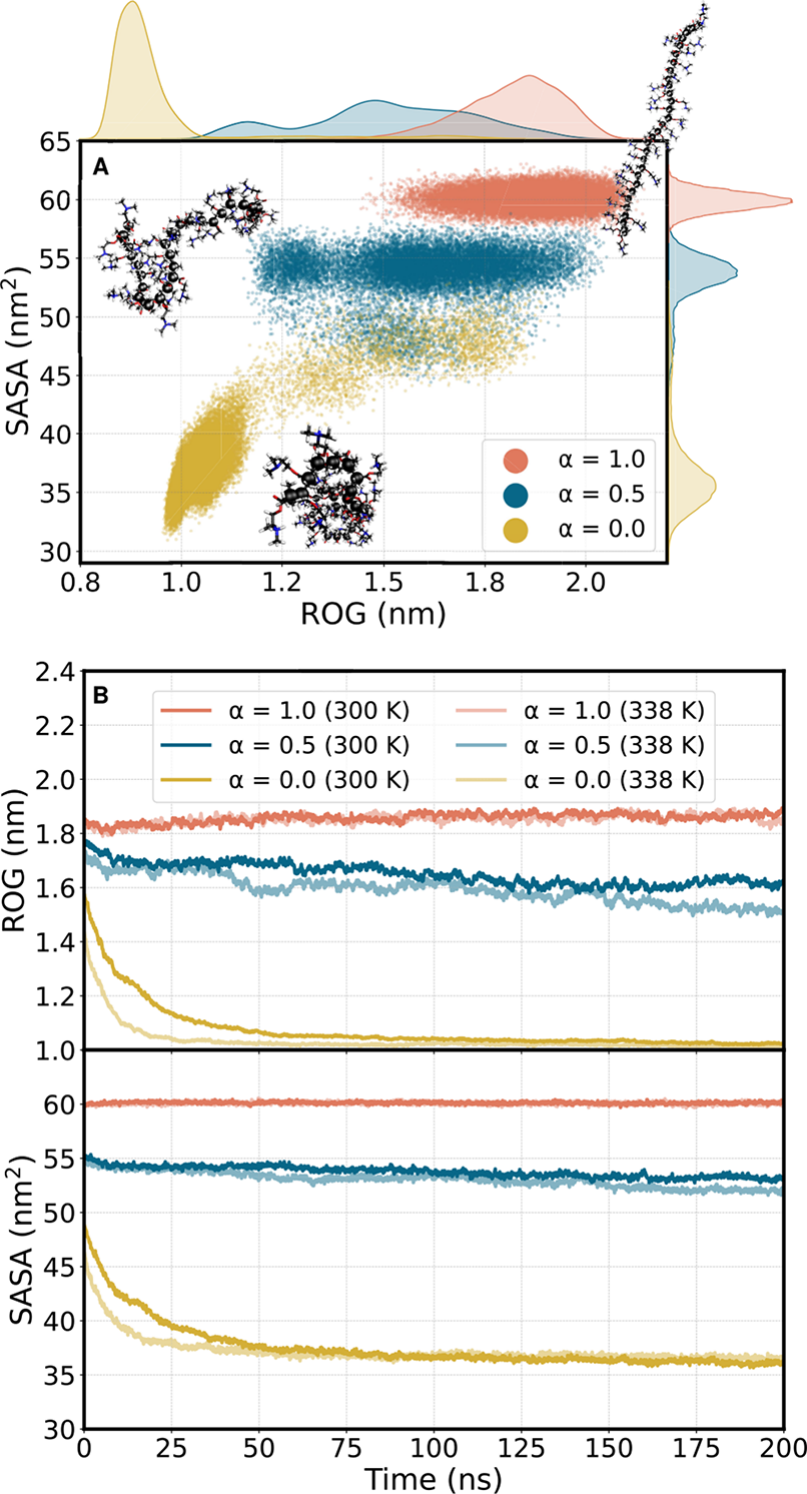
(A) Simulation trajectories taken from the last 100 ns at all protonation degrees α (1.0, 0.5, and 0.0) of individual p(DMAEMA) threads projected into the conformational space defined by the radius of gyration (ROG) versus the solvent-accessible surface area (SASA). The kernel density estimation for both ROG and SASA is displayed along the respective axis edges. Representative structures of each family are shown as insets. Visualization is done using the molecular viewer VMD.^55^ (B) ROG (nm) and SASA (nm^2^) plotted against the entire simulation time of 200 ns for all protonation degrees α (1.0, 0.5, and 0.0) of individual p(DMAEMA) threads at two temperatures, 300 K (dark-) and 338 K (light colors). The plotted values represent the averages calculated from all individual MD simulations for polymer threads for each protonation degree.

Figure 6A shows the SASA plotted against the ROG for all 16 generated protonated, unprotonated, and semiprotonated p(DMAEMA) chains obtained from the last 100 ns of a 200 ns MD simulation, respectively. The kernel density estimators are displayed on the axes.^53^ The first half of the simulation was excluded from this analysis to ensure that the system was properly equilibrated. As it can be seen, for a fully protonated chain, (α = 1.0), the SASA remains relatively constant at around 60 nm^2^. In contrast, the ROG shows more variation, ranging between 1.5 and 2.1 nm. Overall, the highest values for SASA and ROG were observed for fully protonated chains. This indicates that extended polymer strands are the result of the repelling interactions of the protonated monomer units, in agreement with previous studies on p(DMAEMA),^29^ see a representative structure as an inset. Upon protonation of half of the units (α = 0.5), the polymer strands reduce the water accessibility, decreasing the SASA to around 55 nm^2^, while the ROG adapts a range of around 1.2 to 2.0 nm. Both metrics show an increase in variance. Finally, unprotonated chains (α = 0.0) exhibit the least dispersion, with a distinct peak of the SASA at around 36 nm^2^ and the ROG at 1.0 nm —indicative of a globule-like conformation (see inset). A few outliers were observed where the chain gets further extended. The sharp peaks observed in the ROG and SASA of the unprotonated threads suggest that a unique conformational arrangement, the globule-like conformation, is preferably formed. This is in contrast to the partially and fully protonated threads, which fluctuate between partially unfolded and extended structures (see the insets). Overall, we notice that the “globule state” does not correspond to a single molecular conformation, but structures with a specific ROG can have quite a different SASA – similar to what was shown in poly(*N*-isopropylacrylamide).^54^

Additionally, we investigated the temperature dependence of the time-resolved ROG and SASA through the MD simulations for all three (fully protonated, unprotonated, and partially protonated) protonation states by performing simulations at 300 and 338 K (Figure 6B), with these temperatures chosen based on prior theoretical studies.^28,29^ A clear temperature dependence is observed only for a protonation degree of α = 0.5, resulting in a decrease in the ROG by about 0.1 nm at the end of the simulation when the temperature is increased. Unprotonated threads show a faster decrease in ROG and SASA during the first half of the simulation, eventually reaching the same values as in the simulation at 300 K. However, in the fully protonated cases, there are no significant differences between the two temperatures at acidic pH. These findings are in good agreement with the work by Schacher et al., showing the temperature and pH responsiveness of PS-*b*-p(DMAEMA).^17^

**Figure 7:**
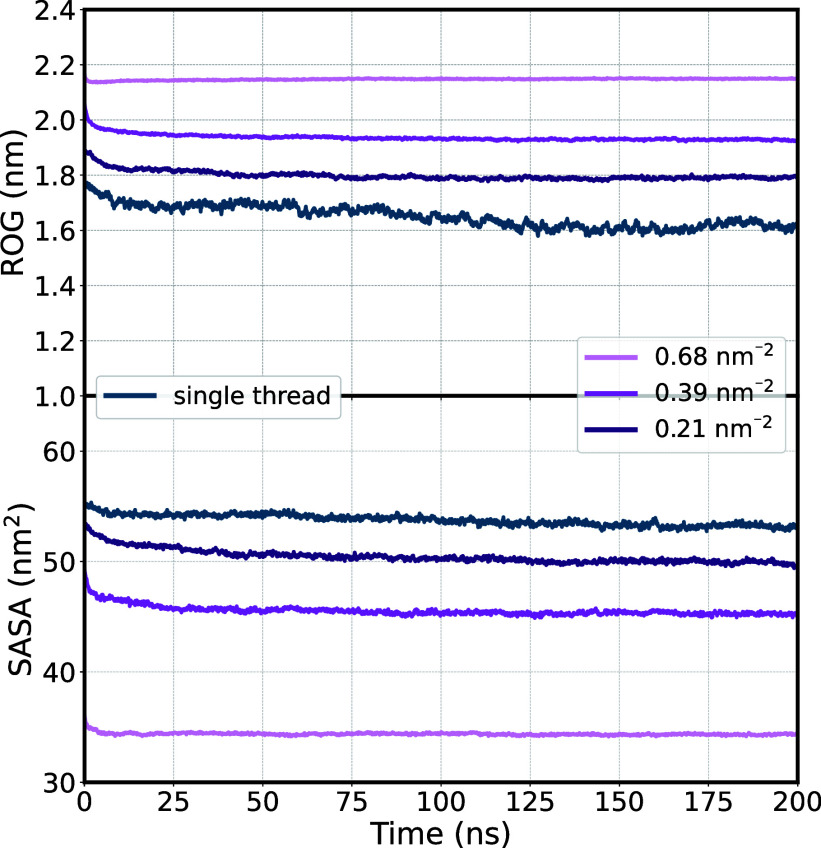
ROG and SASA plotted against the entire simulation time of 200 ns for various grafting densities (0.68, 0.39, and 0.21 nm^–2^) and the single polymer thread with a protonation degree of 0.5, respectively.

Next, we explored how confining individual p(DMAEMA) threads in a restricted space affects their conformational and dynamic properties. First, we compared the ROG and SASA of the polymer brushes of varying grafting densities (σ = 0.68, 0.39, and 0.21 nm^–2^) with the single threads. For that, we averaged the values of all 16 threads of the polymer brush. For the single threads, we averaged the values from all individual simulations with a protonation degree of α = 0.5. The time evolutions of both metrics are depicted in Figure 7. As the grafting density increases, the ROG also rises because there is less space available for the individual strands to coil up. Single threads showed the lowest ROG, coiling up the most due to the absence of steric hindrance from neighboring threads. The highest grafting density resulted in a large and steady ROG over the course of 200 ns simulation. In contrast, lower grafting densities show a slight decrease within the first 25 ns, followed by stable behavior. Unlike the polymer brushes, a single polymer strand exhibits more flexibility, resulting in a noisier profile and equilibrium not being reached before 150 ns of simulation. This highlights the effect of the confinement on the individual polymer threads. Opposite to the ROG, the SASA decreases with increasing grafting density because the water molecules are “pushed out” of the polymer brush, leaving less room for the solvent molecules to fit between the polymer strands. Similarly to the ROG, the highest grafting density resulted in a stable SASA throughout the entire MD simulation. For the single threads, the ROG decreased by approximately 10%, whereas the SASA decreased by only about 2% from the beginning to the end of the simulation. This indicates that spatial confinement has a greater influence on the ROG compared to the SASA. Additionally, this can be attributed to the fact that conformations with varying ROGs can exhibit the same SASA, as previously discussed.

We were also interested in examining the distances between individual p(DMAEMA) threads when they were confined in a polymer brush. Figure S3 shows how these distances evolve during the MD simulation. Lower grafting densities allow for more flexibility, enabling the polymer threads to come closer. A high grafting density, on the other hand, exhibits low flexibility, as indicated by a small decrease in the distance between individual p(DMAEMA) threads. This correlation between grafting density and flexibility is also reflected in the root-mean-square deviation of the respective polymer brushes (see Figure S4). At a high grafting density, the root-mean-square deviation from the starting structure over the course of 200 ns simulation indicates a strong restriction in configurational freedom due to steric hindrance.

#### Radial Distribution Functions (RDFs) and Solvent Distribution

**Figure 8:**
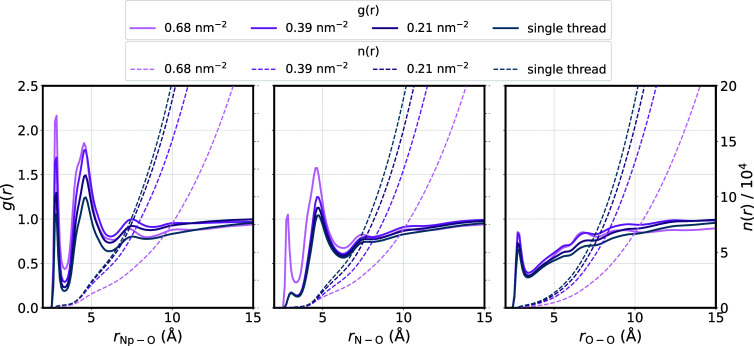
Radial distribution functions (RDFs) of protonated nitrogen atoms (N_p_), unprotonated nitrogen atoms (N), and carbonyl oxygen atoms (O_c_) to water oxygen atoms (O) for different polymer grafting densities and single p(DMAEMA) threads. The running number integral n(r) is given as dashed lines, while solid lines represent the RDF g(r).

The influence of confinement within the polymer and the impact of the local solvent environment are investigated with RDFs between the solute and oxygen atoms of water. Specifically, the RDFs were calculated between the protonated or unprotonated nitrogen atoms or the carbonyl oxygen atoms and the oxygen atoms of water within both the polymer and the individual threads. These results are shown in Figure 8, along with the running number integral n(r), which provides insight into the number of water molecules present in the solvation shells.

The RDFs of the protonated nitrogen atoms show the overall highest intensity, indicating that most water molecules are localized in close vicinity to the protonated nitrogen atoms due to the formation of hydrogen bonds that produce sharp, intense peaks at around 2.8 Å, forming the first solvation shell. A second solvation shell is observed at around 4.6 Å. In contrast, for unprotonated DMAEMA units, only a minor shoulder peak is observed at a distance of 2.8 Å, except in the case of the densest packed polymer brush, which provides less space for water molecules to diffuse between the polymer threads. These findings are supported by the running number integral, which approximately doubles in the first solvation shell upon transitioning from unprotonated to protonated nitrogen atoms. A second solvation shell is again observed at around 4.6 Å. The RDFs of the carbonyl oxygen atoms also reveal a distinct first solvation shell at 2.8 Å, indicating hydrogen bond formation. However, the intensity is significantly lower compared to protonated nitrogen atoms, highlighting a preferred accumulation of water molecules around the protonated nitrogen atoms. Overall, the RDFs lose intensity with decreasing grafting density, reaching a minimum in the case of single threads. This is in good accordance with the insights obtained from the ROG and SASA calculations. A less densely packed polymer brush allows for more diffusion of water molecules. The number of water molecules, the running integral n(r), follows the opposite trend, further supporting the assumption that a high grafting density pushes water molecules out of the polymer brush and into the bulk. Additionally, when the individual threads have greater conformational flexibility, their tendency of coiling up results in water being shielded away from the DMAEMA side chains, hence decreasing the possibility for the formation of hydrogen bonds. In addition to studying the interactions between water molecules and the p(DMAEMA) brushes, we also explored the interactions between the p(DMAEMA) threads and the chloride counterions. Figure S5 shows the RDFs between Cl^–^ and the protonated and unprotonated DMAEMA units, respectively. Similar to the RDFs of water, it can be noticed that as the grafting density decreases, the intensity of g(r) also decreases. Interestingly, the running number integral reveals a trend opposite to that observed for water. This suggests that strong electrostatic interactions between chloride ions and DMAEMA units, especially the protonated ones, reduce the tendency of chloride ions to diffuse out of the brush, effectively trapping the counterions within the polymer brush, particularly the most tightly grafted one.

**Figure 9:**
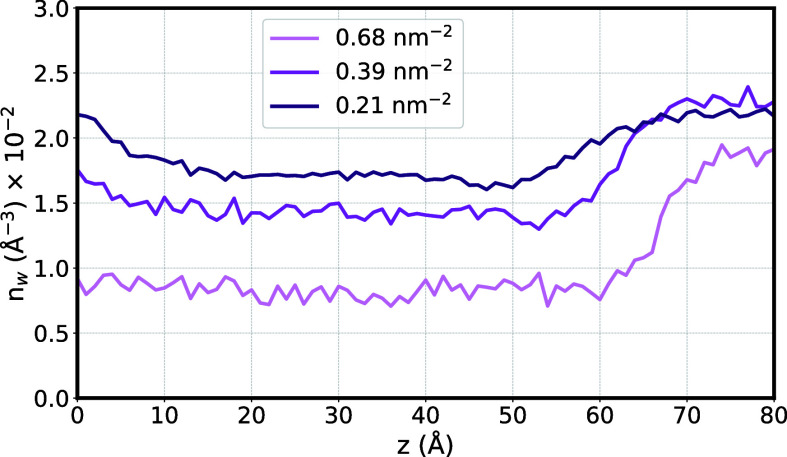
Number density of water molecules, *n*_w_, per Å^3^ plotted against the polymer brush height, *z*, for various grafting densities.

Based on the results from the RDFs, we attributed the lower intensities of the *g*(*r*) in tighter grafted polymer brushes to the effect of water being pushed out into the bulk. To validate this assumption, we calculated the water number density along the polymer brush’s height profile, averaged over the last 50 ns of simulation time. A bin size of 1 Å was chosen for this calculation, and the number of water molecules per volume element of each bin was counted (see Figure 9). Each p(DMAEMA) polymer thread has a height of approximately 60 Å, beyond which we enter the bulk region. As the grafting density increases, the number density of water decreases, further supporting the assumption that confinement causes water to be pushed out into the bulk. In all simulations, it is observed that the number of water molecules increases at the top of the polymer brush (below 60 Å). This indicates greater flexibility at the polymer’s surface due to the opening up of the individual threads. The tighter the polymer brush is packed, the less flexible it becomes, causing the number of water molecules to increase over a greater distance (around 57 Å for the highest grafting density). In the bulk, the number of water molecules per volume element converges to approximately *n*_w_ = 2 × 10^–2^ Å^–3^. Furthermore, as the polymer brush becomes more tightly packed, the increase in water molecules is steeper, resulting in a more pronounced separation between the bulk and brushes.

## Conclusions

This paper presents a comprehensive computational protocol for constructing polyelectrolyte polymer brushes, using poly(*N*,*N*-dimethylaminoethyl methacrylate) (p(DMAEMA)) as a representative case study. Our approach introduces a non-grid-based method for arranging polymer strands, allowing for a natural and flexible spatial configuration that closely mimics the randomness and variability of experimental brush-like structures. Furthermore, we propose a procedure to implement periodic boundary conditions using the PACKMOL software, facilitating the setup of initial geometries for asymmetric polymer brushes in MD simulations. This setup ensures an accurate representation of the interfacial properties and dynamics of these complex systems. Importantly, this protocol is highly adaptable and can be easily applied to construct a wide variety of polymer brushes, providing a robust tool for researchers exploring polyelectrolyte materials in MD simulations.

Using our fully atomistic description, MD simulations are used to investigate the influence of protonation, temperature, and grafting density on the structural and dynamic properties of the polymer brushes and single p(DMAEMA) threads. We found that protonation degrees of α = 0.0 (unprotonated) and 1.0 (fully protonated) showed no temperature dependence, while partially protonated p(DMAEMA) strands exhibited a coil-to-globule transition upon heating. Further, fully protonated/unprotonated p(DMAEMA) threads showed less variance in the radii of gyration as well as SASAs compared to the partially protonated polymer due to electrostatic interactions between the side chains. Upon confinement of the individual polymer threads into a polymer brush, their flexibility is reduced, as expressed by a decrease in the SASA and an increase in the ROG. RDF analysis provided insights into the local solvent environment, revealing that higher grafting densities led to water molecules being pushed out of the polymer brush into the bulk.

We foresee that this flexible workflow for setting up polymer brushes with customizable configurations will facilitate the study of structural, dynamic, and interfacial properties of polyelectrolyte materials across a range of applications. Future work will focus on embedding polyoxometalate catalysts within these brush-like structures to enhance their catalytic properties and broaden their functional applications, improving the understanding of hybrid systems for heterogeneous catalysis.

## Supporting Information Available

Partial charges of p(DMAEMA) used for the MD simulations; root-mean-square deviations providing insights into conformational freedom inside the brushes; RDFs between the counterions and polymer threads illustrating the effect of protonation on the chemical environment; and distances between p(DMAEMA) threads within polymer brushes at varying grafting densities as a measure of thread flexibility (PDF)
Input files and scripts to apply our computational protocol provided in separate Supplementary files (ZIP)
