# Supplementary material for: All-Atom Molecular Dynamics Simulations of Grafted Poly(N,N-dimethylaminoethyl methacrylate) Brushes
Source: J Phys Chem B. 2025 Feb 10;129(7):2105–14. doi: 10.1021/acs.jpcb.4c07928 (PMC11848925; doi:10.1021/acs.jpcb.4c07928)
Supplement: Supplementary file 1 — jp4c07928_si_001.pdf [file jp4c07928_si_001.pdf]

Supporting Information:
All-Atom Molecular Dynamics Simulations of Grafted Poly(N,N-dimethylaminoethyl methacrylate) brushes

Simon Tippner [1,2], David Hernández-Castillo [1,2,3], Felix H. Schacher [4] and Leticia González [1,5]

[1] Institute of Theoretical Chemistry, Faculty of Chemistry, University of Vienna, Währinger Str. 17, 1090 Vienna, Austria.
[2] University of Vienna, Vienna Doctoral School in Chemistry (DoSChem), Währinger Str. 42, 1090 Vienna, Austria.
[3] Present address: University Duisburg-Essen, Faculty of Chemistry, Theoretical Catalysis and Electrochemistry, Universitätsstraße 5, 45141 Essen, Germany.
[4] Laboratory of Organic and Macromolecular Chemistry (IOMC), Friedrich Schiller University Jena, Lessingstraße 8, 07743 Jena, Germany.
[5] Vienna Research Platform on Accelerating Photoreaction Discovery, Währinger Str. 17, 1090 Vienna, Austria.

Contents

S1 Scanning electron microscope measurements S2

S2 Partial charges of p(DMAEMA) S2

S3 Distances S4

S4 Root mean square deviation (RMSD) S5

S5 Radial distribution function (RDF) of chloride ions S6

S1 Scanning electron microscope measurements

Scanning elctron microscope images were taken on a Leo Gemini 1530. The specimens were dried under vacuum overnight and coated with approximately 2-nm Pd. For cross-section, a piece of the sample was frozen together with the sample holder and broken afterwards.

S2 Partial charges of p(DMAEMA)

Figure S1 displays the atom labeling of a p(DMAEMA) unit which are needed to assign the Coulomb partial charges q (e) to the respective atoms.

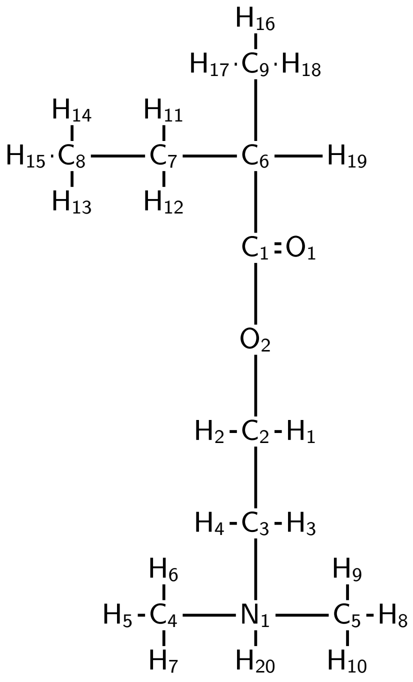

Figure S1: Atom labeling of a DMAEMA unit.

	q (e)			
Atom	DMAEMA (R) unprotonated	DMAEMA (S) unprotonated	DMAEMA (R) protonated	DMAEMA (S) protonated
C7	0.14	0.13	0.12	0.12
H11	-0.02	-0.01	-0.00	-0.00
H12	-0.02	-0.01	-0.00	-0.00
C6	-0.03	-0.03	-0.04	-0.04
C1	0.78	0.77	0.74	0.74
O1	-0.56	-0.56	-0.50	-0.50
O2	-0.54	-0.54	-0.49	-0.49
C2	0.35	0.35	0.21	0.20
C3	-0.01	-0.01	-0.16	-0.16
N1	-0.33	-0.34	-0.08	-0.08
C4	-0.05	-0.05	-0.36	-0.36
H5	0.05	0.05	0.19	0.19
H6	0.05	0.05	0.19	0.19
H7	0.05	0.05	0.19	0.19
C5	-0.05	-0.05	-0.36	-0.36
H8	0.05	0.05	0.19	0.19
H9	0.05	0.05	0.19	0.19
H10	0.05	0.05	0.19	0.19
H20	/	/	0.36	0.35
H3	0.05	0.04	0.16	0.16
H4	0.05	0.04	0.16	0.16
H1	-0.00	-0.00	0.05	0.05
H2	-0.00	-0.00	0.05	0.05
C9	-0.27	-0.26	-0.26	-0.26
H16	0.07	0.07	0.09	0.09
H17	0.07	0.07	0.09	0.09
H18	0.07	0.07	0.09	0.09

Table S1: Coulomb partial charges q (e) of all four DMAEMA monomers (R, S (unprotonated/protonated)) obtained with the RESP charge fitting method.

Table S1 shows the Coulomb partial charges q (e) of all four DMAEMA monomers (R, S (unprotonated/protonated)) obtained with the RESP charge fitting method.

S3 Distances

Figure S2 presents the average and minimal distances between p(DMAEMA) threads within polymer brushes of varying grafting densities at different stages of the MD simulation, as described in the *Computational Details*.

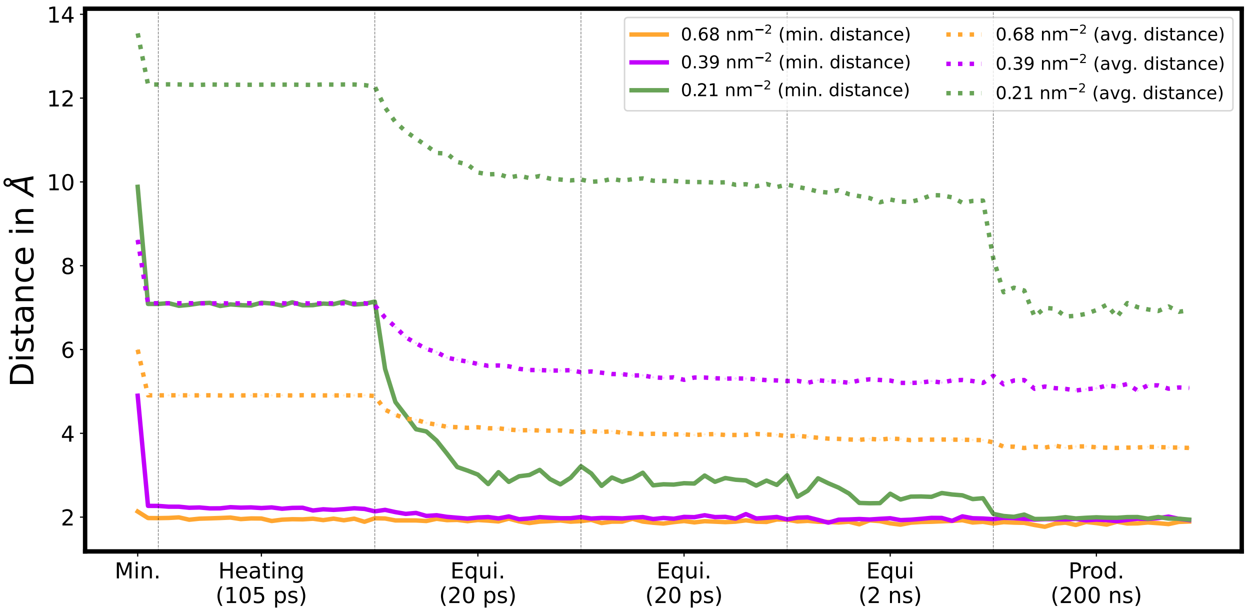

Figure S2: Minimal (solid lines) and average (dashed lines) distances between p(DMAEMA) threads for varying grafting densities, respectively.

The first value after minimization represents the initial structure, as only the solvent was minimized at this stage. The second value corresponds to the fully minimized system. In all cases, a decrease in distances was observed due to attractions between p(DMAEMA) threads. During the heating process, the system remained static since the solute was fixed. However, once the restraints were removed during the equilibration and production steps, a continuous overall decrease in distance was observed. For the polymer brush with a grafting density of 0.68 nm^{-2}, no decrease in the minimal distance was noted, indicating that steric hindrance prevented the polymer threads from coming any closer. In contrast, brushes with medium and low grafting densities exhibited minimal distances as small as 2 Å, suggesting greater flexibility.

S4 Root mean square deviation (RMSD)

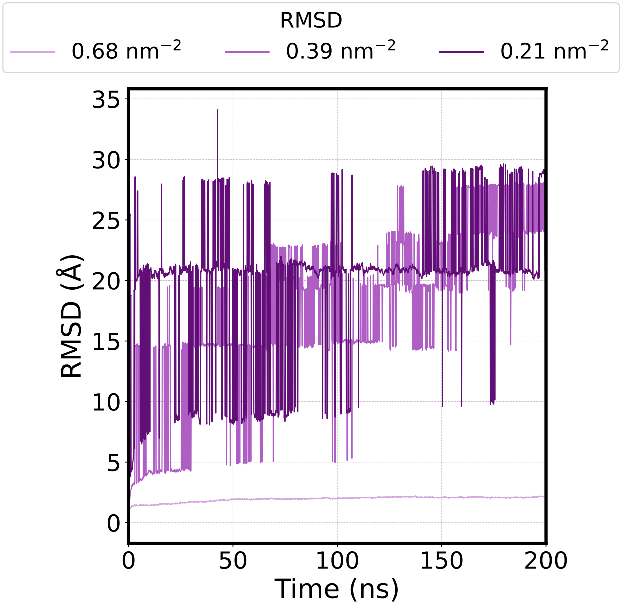

Figure S3: RMSDs plotted against the simulation time for polymer brushes at different grafting densities.

S5 Radial distribution function (RDF) of chloride ions

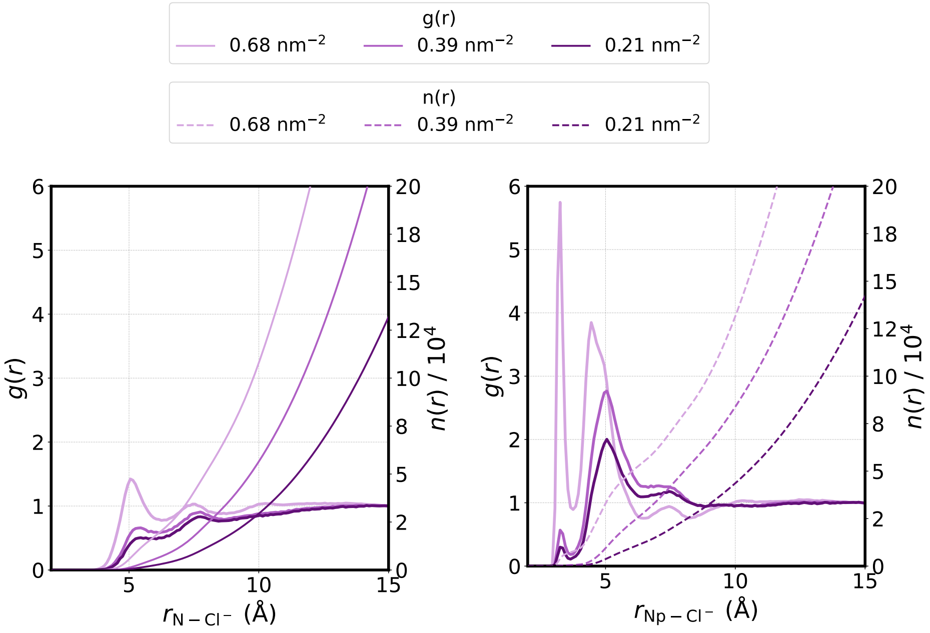

Figure S4: RDFs of protonated nitrogen atoms (N_p) and unprotonated nitrogen atoms (N) to chloride ions (Cl^-) for different polymer grafting densities. The running number integral $n(r)$ is given in dashed lines, while solid lines represent the RDF $g(r)$.
